# Association between the PNPLA3 I148M Polymorphism and Non-Alcoholic Fatty Liver Disease in the Uygur and Han Ethnic Groups of Northwestern China

**DOI:** 10.1371/journal.pone.0108381

**Published:** 2014-10-07

**Authors:** Yuexin Zhang, Wen Cai, Jiangmei Song, Lei Miao, Bei Zhang, Qin Xu, Lijuan Zhang, Hua Yao

**Affiliations:** 1 Center of Infectious Diseases, First Affiliated Hospital of Xinjiang Medical University, Urumqi, China; 2 School of Nursing, Xinjiang Medical University, Urumqi, China; 3 School of Public Health, Xinjiang Medical University, Urumqi, China; 4 School of Basic Medical, Xinjiang Medical University, Urumqi, China; 5 First Affiliated Hospital of Xinjiang Medical University, Urumqi, China; The University of Hong Kong, Hong Kong

## Abstract

**Objective:**

Multiple common gene variants play a role in non-alcoholic fatty liver disease (NAFLD) susceptibility. Our goal was to investigate the association between variants polymorphisms and NAFLD in the Uygur and Han from Northwestern China.

**Methods:**

Eight tag single nucleotide polymorphisms (tSNPs) previously reported to be associated with NAFLD were characterized in 396 NAFLD individuals and 399 controls. The association of variants with NAFLD in the Uygur and Han was assessed using the chi-squared (χ^2^) test in different gene models. Unconditional logistic regression analysis was performed to obtain the odds ratios (ORs) for risk of NAFLD and their 95% confidence intervals (CI), adjusted for confounding factors. Finally, stratified analysis was used to explore the potential gene-environment interactions on the risk of NAFLD.

**Results:**

In a recessive model, we found a potential association between rs738409 and NAFLD in both ethnic groups: Chinese Han (OR = 1.84, 95% CI: 1.03–3.27, *p* = 0.036), Uygur (OR = 2.25, 95% CI: 1.23–4.09, *p* = 0.006). The multiple logistic regression revealed that *PNPLA3 rs738409* GG genotype may increase the risk of NAFLD by adjusting some confounding factors: Han (OR = 5.22, 95% CI: 1.94–14.04, *p* = 0.001), Uygur (OR = 4.29, 95% CI: 1.60–11.48, *p* = 0.004). Stratified analysis found that rs738409 polymorphism appeared to have interaction with sex, smoking status in Uygur, and have interaction with sex, age, BMI stage, lifestyle in Han.

**Conclusion:**

Our data suggest the PNPLA3 I148M polymorphism influences susceptibility to NAFLD in the Han and Uygur of Northwestern China.

## Introduction

Non-alcoholic fatty liver disease (NAFLD) is the histological expression of various assaults on the liver, including obesity as well as lipid and glucose abnormalities [1]. Defined by an excess of liver triglycerides in the absence of excessive alcohol intake or exposure to toxins, NAFLD has become a major cause of liver disease worldwide. It can result in non-alcoholic simple fatty liver, non-alcoholic fatty hepatitis, and liver cirrhosis and hepatocellular carcinoma (HCC) [2]. With a prevalence of 20–34%, NAFLD is the most common liver disease in industrialized countries [3], [4]. Although the prevalence of NAFLD is somewhat lower in Asia, the frequency is increasing, and the disorder is now being seen in younger individuals [5], [6]. Susceptibility to NAFLD and its natural history appear to be modulated by gene-environment interactions [1]. To date, a number of studies have found genetic factors that play a significant role in NAFLD pathogenesis, such as *APCO3*, *APOA5*, *MTTP*, *PEMT* and *PNPLA3* gene [7]–[10].

Xinjiang is located on the northwest border of China, where central and western Asian cultures converge. This region is populated by a variety of ethnicities, but primarily by the Uygur. The Uygur have a unique genetic background, lifestyle, and dietary habits. It is not known whether a difference in NAFLD prevalence associated with *APCO3*, *APOA5*, *MTTP*, *PEMT* and *PNPLA3* gene is present between the Uygur and Han populations in Xinjiang. Here, we studied ethnic differences in NAFLD susceptibility related to eight tag single nucleotide polymorphisms in Xinjiang.

## Methods

### Study Population

We performed a 1∶1 frequency-matched NAFLD case-control study based out of a healthy examination center in the First Affiliated Hospital of Xinjiang Medical University, from January 2011 to December 2012. Briefly, the case group consisted of 396 unrelated individual NAFLD patients (193 Han, mean age 43.70±10.72 years, and 203 Uyghur, mean age 42.40±8.79 years) with features of NAFLD and ultrasonographic (US) examinations. The diagnosis of NAFLD was performed under standard clinical evaluation conditions according to the AASID criteria [11]. Exclusion criteria included: missing covariate information; alcohol consumption greater than 20 g/day for males or 10 g/day for females; a positive test for hepatitis B antigens or hepatitis C antibodies; history of other known causes of chronic liver disease, such as viral hepatitis or autoimmune hepatitis; use of hepatotoxic medications; and history of cancer, respiratory ailments, renal diseases, or endocrine disorders. Additionally, 399 healthy individuals (211 Han, mean age 42.90±10.00 years, and 188 Uyghur, mean age 41.60±9.07 years) were chosen to serve as controls; they were selected randomly during the same study period, from local residents who underwent routine health checks and were free of any known major diseases. Controls were frequency-matched to the NAFLD patients according to sex, age, ethnicity, and area of residence.

### Ultrasonographic examination

Liver US scanning performed to assess the degree of steatosis. All US were performed by the same operator who was blinded to laboratory values using Siemens CDUS512 apparatus equipped with a convex 3.5 MHz probe. Diagnosis of NAFLD was based on the presence of an ultrasonographic pattern consistent with “bright liver”(brightness and posterior attenuation) with stronger echoes in the hepatic parenchyma than in the renal parenchyma, vessel blurring, and narrowing of the lumen of the hepatic veins in the absence of findings suggestive of other chronic liver diseases.

### Demographic and Clinical Data

Each participant underwent an anthropometric assessment, which including measurement of weight, stature, and waist circumference (WC). Body mass index (BMI) was calculated as weight (kg)/stature (m^2^). Blood pressure was measured using an automatic clinical blood pressure monitor while subjects were seated comfortably after a rest period of at least 10 min. All blood samples were obtained from the antecubital vein in the morning, after an overnight fast. Serum triglycerides (TGs), total cholesterol (TC), high-density lipoprotein (HDL), low-density lipoprotein (LDL), serum uric acid (SUA), fasting plasma glucose (FPG), aspartate aminotransferase (AST), alanine aminotransferase (ALT), blood urea nitrogen (BUN), and serum creatinine (SCr) were measured.

According to the clinical diagnostic criteria of Hepatology fatty liver and alcoholic liver disease, overweight or obese: BMI ≥25.0 kg/m^2^, High blood glucose ≥6.1 mmol/L, High blood pressure: systolic blood pressure/diastolic blood pressure ≥140/90 mmHg, Serum triglycerides (TGs) ≥1.7 mmol/L, high-density lipoprotein (HDL) <0.9 mmol/L in male and <0.9 mmol/L in female, Cholesterol ≥5.18 mmol/L, high uric acid >420 µmol/L (men and postmenopausal women), and premenstrual women >350 µmol/L.

### Ethics Statement

The use of human blood samples and the study protocol conformed to the principles expressed in the Declaration of Helsinki and were approved by the institutional ethics committee of the First Affiliated Hospital of Xinjiang Medical University. Written informed consent was obtained from all participants prior to participation in the study.

### Genotyping

Genomic DNA was extracted from whole-blood samples collected in EDTA tubes following standard procedures and stored at −20°. Approximately 10 ng DNA was used for genotyping via SnaPshot PCR (Applied Biosystems, ABI, USA), according to the manufacturer’s directions. Total reaction volume was 20 µl, and each reaction contained 1x HotStarTaq buffer, 3.0 mM Mg^2+^, 0.3 mM dNTPs, and 1 U HotStarTaq polymerase (Qiagen Inc, Beijing, China). The cycling conditions were as follows: 95°C for 2 min, 11 cycles of 94°C for 20 s, 65°C for 40 s, and 72°C for 90 s; 24 cycles of 92°C for 20 s, 59°C for 30 s, and 72°C for 90 s; 72°C for 2 min; followed by storage at 4°C. Subsequently, PCR products were purified by adding 1 U shrimp alkaline phosphatase (SAP) and 1 U exonuclease I to each reaction; the mixtures were incubated in a 37°C water bath for 1 hour and inactivated at 75°C for 15 min.

Multiple single-base extension reactions were carried out. The extension reaction system (10 µl) included 5 µl SNaPshot Multiplex Kit (Applied Biosystems), 2 µl multiple purified PCR products, 1 µl 0.8 µM extension primer mixture, and 2 µl ultrapure water. The PCR conditions were as follows: 96°C for 1 min; 28 cycles of 96°C for 10 s, 52°C for 5 s, and 60°C for 30 s; then storage at 4°C. Subsequently, PCR products were purified by adding 1 U SAP to each reaction; the mixtures were incubated in a 37°C water bath for 1 hour and inactivated at 75°C for 15 min. Purified extension products were sequenced and typed using an ABI3130XL sequencer (Applied Biosystems).

### Statistical Methods

All continuous data are presented as means ± standard deviations (SDs). Pearson’s x^2^ test and T-test were used to compare the distribution of categorical variables and continuous variables, respectively. Differences in continuous variables among the subjects with three genotypes of related genes were assessed using the ANOVA.

Each SNP frequency in control subjects was tested for departure from Hardy–Weinberg Equilibrium (HWE). We calculated the genotype frequencies of case and control subjects using the chi-square test. Odds ratio (OR) and 95% confidence intervals (CIs) were calculated by unconditional logistic regression. To eliminate the influence of confounding factors, we further adopt multiple logistic regression analysis adjusted for confound factors to evaluate the effects of the polymorphism on the risk of cancer. The Backward method based on Likelihood Ratio Test was used to select the variable on NAFLD. Finally, stratified analysis was used to explore potential gene-environment interactions. Enter the product of single risk factor and gene polymorphism as a single term into the model to evaluate the interaction.

Statistical analyses were performed using Microsoft Excel and the SPSS 19.0 statistical package (SPSS, Chicago, IL). And two-sided *p-*values<0.05 were considered statistically significant.

## Results

The SNP information, observed allele frequencies and Hardy-Weinberg test result are presented in Table 1. Table 1 shows the results of the test investigating departure from Hardy-Weinberg equilibrium (HWE) in controls: a few results were <0.10 and one was 0.05.

**Table 1:** Basic information on candidate tSNPs for NAFLD.

SNP	Gene	Minor allele	Major allele	Band	HWE * *p*		Allele ORs (95% CI)	Pearson Chi-Square** *p*-value
					Han	Uygur		
rs1800591	*MTTP*	T	G	4q23	0.328	0.870	0.978 (0.757–1.263)	0.863
rs3816873	*MTTP*	C	T	4q23	0.376	0.830	0.938 (0.728–1.209)	0.621
rs2854116	*APOC3*	C	T	11q23.3	0.992	0.939	1.015 (0.833–1.237)	0.884
rs3135506	*APOA5*	C	G	11q23.3	-	0.182	1.139 (0.565–2.297)	0.716
rs662799	*APOA5*	G	A	11q23.3	0.081	0.081	1.085 (0.859–1.372)	0.494
rs6006460	*PNPLA3*	T	G	22q13.31	-	-	-	-
rs738409	*PNPLA3*	G	C	22q13.31	0.322	0.945	1.501 (1.224–1.841)	< 0.001
rs7946	*PEMT*	T	C	17p11.2	0.05	0.190	1.131 (0.915–1.397)	0.257

HWE: Hardy-Weinberg equilibrium.

*p<0.1 indicates statistical significance.

**p<0.05 indicates statistical significance.

Significant difference (*p*<0.05) were found between patient with NAFLD and controls, in terms of BMI, WC, SBP, DBP, HBG, FBG, TG, TC, LDL, SUA, AST, ALT and HLD in Uygur and Han populations, respectively. As shown in Table S1, the mean BMI, WC, SBP, DBP, HBG, FBG, TG, TC, LDL, SUA, AST, ALT were significantly higher in both Uygur and Han NAFLD cases than controls. In addition, the level of HDL was significantly lower in subjects with NAFLD than controls.

We used various genetic models to investigate the association between the PNPLA3 I148M polymorphism and NAFLD, as shown in Table 2. A recessive model revealed a potential association with NAFLD in both the Han and Uygur samples: Chinese Han (OR = 1.84, 95% CI: 1.03–3.27, *p* = 0.036), Uygur (OR = 2.25, 95% CI: 1.23–4.09, *p* = 0.006).

**Table 2:** Variable genetic models of the association between the PNPLA3 I148M polymorphism (rs738409) and NAFLD in the Uygur and Han of Northwestern China.

Model	Genotype	Uygur		Han	
		OR (95% CI)	p-value	OR (95% CI)	p-value
Codominant	C/C	1	0.009*	1	0.040*
	G/C	1.37 (0.89–2.11)		1.37 (0.89–2.10)	
	G/G	2.64 (1.39–5.00)		2.20 (1.18–4.13)	
Dominant	C/C	1	0.022*	1	0.044*
	G/C-G/G	1.60 (1.07–2.40)		1.52 (1.01–2.29)	
Recessive	C/C-G/C	1	0.006*	1	0.036*
	G/G	2.25 (1.23–4.09)		1.84 (1.03–3.27)	
Log-additive	---	1.55 (1.16–2.08)	0.009*	1.45 (1.08–1.95)	0.012*

* *p*<0.05 indicates statistical significance.

After adjusted for confounding factors (sex, age, BMI Stage, BP Stage, GLU Stage, TG Stage, TC Stage, LDL Stage, Hyperuricemia, Smoking Status, Life style, Family History Hypertension, Family History Diabetes, Family History of CHD, CHD, Family History of Cancer), the fitted final multiple logistic regression model revealed that, the variant PNPLA3 rs738409 genotypes CG and GG were independent risk factors for NAFLD (Table 3). Specifically, compared with the subjects with the homozygous CC alleles, the odds of having NAFLD would be 4.29-fold (95% CI, 1.60–11.48, *p* = 0.004) and 5.22-fold (95% CI, 1.94–14.04, *p* = 0.001) with homozygous GG alleles, respectively in Uygur and Han.

**Table 3:** Association between *PNPLA3* and risk of NAFLD based on multiple logistic regression analysis, adjusted for confounding factors.

Variable	Uygur			Han		
	OR	95% CI	*p*-value	OR	95% CI	*p*-value
Sex						
female	1	-		-	-	
male	3.22	1.54–6.73	0.002	-	-	-
Age (years)			0.002			0.033
≤40	1	-	-	-		-
41–59	0.25	0.11–0.55	0.001	0.79	0.43–1.45	0.454
≥60	0.2	0.04–1.12	0.067	0.23	0.07–0.69	0.009
BMI						
<25 kg/m^2^	1	-				
≥25 kg/m^2^	15.46	5.70–41.93	0	11.25	6.01–21.06	< 0.000
BP Stage						
<140/90 mmHg	1	-		1	-	
≥140/90 mmHg	2.44	1.21–4.92	0.012	1.96	1.05–3.68	0.036
Glucose Stage						
<6.1 mmol/L	1	-		-	-	
≥6.1 mmol/L	7.48	1.49–37.49	0.014	-	-	-
TG Stage						
<1.7 mmol/L	1	-		1	-	
≥1.7 mmol/L	4.73	2.26–9.92	<0.000	4.13	2.25–7.58	< 0.000
Family History ofHypertension						
No	1	-		-	-	
Yes	0.46	0.23–0.89	0.022	-	-	-
rs738409			0.012			0.005
CC genotype	1	-		1	-	-
GC genotype	1.08	0.56–2.12	0.812	1.42	0.77–2.64	0.266
GG genotype	4.29	1.60–11.48	0.004*	5.22	1.94–14.04	0.001*

*p<0.05 indicates statistical significance.

We also observed significant differences in Serum uric acid level was seen among the 3 genotype in both Uygur and Han. SBP and DBP were significantly different in Uygur with the *PNPLA3* I148M polymorphism. Other clinical parameters did not observe any association (Table S2).

Finally, we explored combined effects of the PNPLA3 polymorphisms and some clinical parameters on the risk of NAFLD. Rs738409 polymorphisms appeared to have a joint effect with sex, smoking status in Uygur ethnic, and have interaction with sex, age, BMI stage, lifestyle in Han ethnic (*p*<0.05). No significant interactions between rs738409 and other clinical parameters on NAFLD risk were observed in Uygur and Han ethnic, respectively (Table 4).

**Table 4:** Combined effects of *PNPLA3* rs738409 polymorphisms and environmental and ‘internal’ exposures on the risk of NAFLD.

		Uygur			Han	
Exposures	genotype			genotype		
	CC	CG	GG	CC	CG	GG
Sex						
female	1	1.60 (0.87–2.92)	1.16 (0.42–3.16)	1	1.58 (0.86–2.90)	7.21 (2.22–23.35)
male	1	1.16 (0.62–2.15)	4.34 (1.78–10.60)	1	1.19 (0.65–2.18)	1.12 (0.49–2.55)
*p*-value for interaction	0.050*			0.026*		
Age						
≤40	1	1.19 (0.65–2.18)	1.12 (0.49–2.55)	1	1.54 (0.78–3.05)	0.79 (0.29–2.16)
41–59	1	1.20 (0.68–2.12)	2.90 (1.29–6.52)	1	1.59 (0.88–2.87)	4.76 (1.89–12.00)
≥60	1	2.00 (0.19–20.61)	1.00 (0.04–24.55)	1	0.23 (0.04–1.30)	---
*p*-value for interaction	0.930			0.002*		
BMI Stage						
<25 kg/m^2^	1	3.68 (0.36–37.14)	16.29 (1.47–179.97)	1	1.86 (0.71–4.86)	2.88 (0.93–8.96)
≥25 kg/m^2^	1	1.34 (0.79–2.28)	2.25 (1.00–5.04)	1	1.52 (0.83–2.79)	---
*p*-value for interaction	0.260			0.022*		
Smoking Status						
No smoking	1	1.33 (0.81–2.19)	3.67 (1.78–7.57)	1	1.22 (0.75–1.99)	1.72 (0.86–3.45)
Occasionally smoking	1	1.47 (0.28–7.63)	---	1	1.60 (0.23–11.27)	---
smoking	1	1.35 (0.46–3.96)	0.24 (0.02–2.34)	1	1.40 (0.47–4.13)	3.50 (0.56–21.81)
Quit smoking	1	---	---	1	---	---
*p*-value for interaction	0.022*			0.240		
Lifestyle						
active	1	1.93 (0.46–8.05)	0.43 (0.04–5.06)	1	0.81 (0.20–3.22)	---
balanced	1	2.31 (1.05–5.11)	5.96 (1.69–20.99)	1	0.54 (0.24–1.24)	1.00 (0.30–3.35)
sedentary	1	1.00 (0.57–1.74)	2.23 (0.98–5.03)	1	2.25 (1.30–3.90)	2.78 (1.28–6.00)
*p*-value for interaction	0.130			0.041*		

*p<0.05 indicates statistical significance.

## Discussion

In this case-control study, we selected 8 tag SNPs in the *PNPLA3* gene and examined these variants on NAFLD in Uygur and Han ethnic groups of Northwestern China population. Our study results indicated that individuals carrying the rs738409 G-allele may predispose them to NAFLD. Compared with subjects with the CC homozygous genotype, carriers of heterozygous CG and homozygous GG genotypes had a significantly increased risk of NAFLD.

*PNPLA3* encodes a 481 amino acid protein with a molecular mass of approximately 53 kDa that is known as adiponutrin. In humans, this gene is primarily expressed in the intracellular membrane fraction of hepatocytes [12]. After feeding, Steroid Regulatory Element Binding Protein-1c, the master regulator of lipogenesis, induces production of *PNPLA3* in the liver; *PNPLA3* is also induced during insulin resistance [13]. The physiological function of *PNPLA3* is not fully understood, but it displays a high level of homology to adipose TG lipase and seems to possess both TG lipase and acyl coenzyme A-independent transacylase activity [14]. In human adipose tissue, *PNPLA3* expression is regulated by energy intake [15].

In past research, the PNPLA3 I148M (tSNP rs738409) polymorphism has been associated with loss of the protein’s hydrolyzing function and triglyceride accumulation [12], which appears to confer greater susceptibility to NAFLD [16], [17]. In recent years, the patatinlike phospholipase domain-containing 3 (*PNPLA3*) gene has been studied in relation to liver steatosis and liver disease outcomes. PNPLA3 is involved in lipid metabolism; it is predominantly expressed in liver and adipose tissue and exerts both lipolytic and lipogenic activity in vitro [18].

The I148M polymorphism was first reported to be associated with hepatic TG content in a genome-wide screen [16]. The I148M polymorphism results in a critical amino acid change next to PNPLA3’s catalytic domain, likely reducing substrate access and reducing enzymatic activity towards glycerolipids, thereby leading to the development of macrovescicular steatosis [12], [19]. Several recent studies have shown that the I148M polymorphism is closely associated with an increase in liver fat content [20], [21]. Romeo et al. have explored whether the polymorphism is associated with hepatic fat content in individuals of different ancestry [16]. Romeo et al. also found that Hispanics had the highest propensity for developing NAFLD, followed by European Americans; African-Americans were the group with the lowest NAFLD risk [22].

In the present study, we found that compared with the rs738409 CC homozygote, the rs738409 CG heterozygote and GG homozygote were associated with a significantly increased risk of developing NAFLD in Uygur and Han Chinese population, which is consistent with the previous report by Li et al. [23]. Ethnic differences in the prevalence of NAFLD have been reported across studies derived from multiethnic patient populations, the highest frequency of the allele was in Hispanics (0.49), with lower frequencies observed in European Americans (0.23) and African-Americans (0.17) [4]. According to the National Center for Biotechnology Information human SNP database, the G allele frequency is 0.34 in Han Chinese, 0.44 in Japanese, 0.22 in Europeans, and 0.12 in Africans. From this perspective, based on the PNPLA3 rs738409 G allele frequency of 43% in Han population and 42% in Uygur ethnic, we estimate that the effect of PNPLA3 rs738409 polymorphisms in Asians will confer a much higher genetic susceptibility to NAFLD than in Europeans and Africans.

This study found that the Waist circumference, BMI and in Uygur were significantly higher than those of the Han, which suggests that obvious overweight and central obesity exist in Uygur parents. This may be a reason for the NAFLD risk of Uygur is higher than Han population. The levels of ATL and AST are higher in NAFLD parents than health people, no matter in Han and Uygur ethnic. We evaluated the changes in ATL and AST, which were used as marker of liver fat accumulation and are common used in clinical practice [24]. But we did not observe significant differences in plasma concentration of these transaminases among wild homozygote genotype, heterozygote genotype and variant homozygote genotype, even when independent of genetic variation in *PNPLA3*. We also found that the G-allele of the PNPLA3 rs738409 SNP increased the susceptibility to NAFLD independent of SUA in Uygur and Han ethnic groups. Liu et al. found SUA levels were independently correlated with NAFLD in Chinese postmenopausal women. SUA levels in the higher quartiles of the normal range may be an independent risk factor of NAFLD [25].

A major strength of study is the comprehensive analysis of polymorphism in PNPLA3 gene and clinical data that may influence the risk of NAFLD. In our analyses, we adjusted for important clinical variables that could confound the effect of the genetic variants on NAFLD. A limitation of the study is also exists. Abdominal ultrasound is widely used for screening fatty liver disease. Ultrasound is very sensitive, but it cannot detect small amounts of hepatic steatosis and it does not provide reliable quantitive information [26]–[28]. Additional, including operator-dependency, an inability to distinguish NASH from other subtypes of NAFLD, and an inability to accurately stage hepatic fibrosis [28].

In conclusion, we were investigated clinical and biochemical characteristics associated with the polymorphism in both the Uygur and Han populations. This study also provides reliable evidence that the PNPLA3 I148M polymorphism is a risk factor for NAFLD in the Han of Northwestern China. In addition, we are the first to report that the same association is present in the Uygur population. Mechanisms underlying this association, and whether PNPLA3 genotype influences NAFLD events need to be further investigated.

## Supporting Information

Table S1 **Clinical and biochemical characteristics of subjects, according to NAFLD status.**
(DOCX)

Table S2 **Clinical and biochemical characteristics of participants, stratified by PNPLA3 I148M polymorphism status.**
(DOCX)
