# Supplementary material for: Association between the PNPLA3 I148M Polymorphism and Non-Alcoholic Fatty Liver Disease in the Uygur and Han Ethnic Groups of Northwestern China
Source: PLoS One. 2014 Oct 7;9(10):e108381. doi: 10.1371/journal.pone.0108381 (PMC4188522; doi:10.1371/journal.pone.0108381)
Supplement: Table S1 — Clinical and biochemical characteristics of subjects, according to NAFLD status. (DOCX) [file pone.0108381.s001.docx]

Table S1 Clinical and biochemical characteristics of subjects, according to NAFLD status

| Variable | Uygur | | *p*-value | Han | | *p*-value |
| --- | --- | --- | --- | --- | --- | --- |
|  | cases (n=203) | controls (n=188) |  | cases (n=193) | controls (n=211) |  |
| BMI (kg/m^2^) | 28.75± 3.90 | 24.67 ± 3.43 | < 0.001* | 27.07 ± 3.96 | 22.79 ± 2.98 | < 0.001* |
| WC (cm) | 98.43 ± 9.11 | 87.82 ± 9.70 | < 0.001* | 93.83 ± 8.92 | 81.54 ± 9.81 | < 0.001* |
| SBP (mmHg) | 123.84 ± 16.14 | 117.19 ± 15.49 | < 0.001* | 125.79 ± 15.56 | 115.25 ± 15.48 | < 0.001* |
| DBP (mmHg) | 77.51 ± 11.82 | 73.49 ± 10.52 | < 0.001* | 80.40 ± 12.30 | 74.42 ± 11.04 | < 0.001* |
| HBG (g/L) | 144.43 ± 16.71 | 140.45 ± 18.73 | 0.034 * | 147.12 ± 13.78 | 141.74 ± 14.94 | 0.001 * |
| FBG (μmol/L) | 5.49 ± 1.93 | 4.83 ± 0.46 | < 0.001* | 5.43 ± 1.14 | 5.09 ± 0.59 | < 0.001* |
| TG (mmol/L) | 2.00 ± 1.65 | 1.22 ± 0.70 | < 0.001* | 2.50 ± 2.39 | 1.57 ± 1.74 | < 0.001* |
| TC (mmol/L) | 4.96 ± 1.06 | 4.69 ± 0.79 | 0.005* | 5.00 ± 0.94 | 4.58 ± 0.83 | < 0.001* |
| HDL (mmol/L) | 1.19 ± 0.32 | 1.38 ± 0.35 | < 0.001* | 1.13 ± 0.34 | 1.28 ±0.38 | < 0.001* |
| LDL (mmol/L) | 3.08 ± 0.77 | 2.85 ± 0.66 | 0.006* | 2.98 ± 0.84 | 2.69 ± 0.65 | 0.001* |
| BUN (mmol/L) | 4.78 ± 1.12 | 4.77 ± 1.24 | 0.899 | 5.08 ± 1.31 | 5.09 ± 1.47 | 0.934 |
| SCr (μmol/L) | 68.67 ± 17.76 | 69.10 ± 19.97 | 0.823 | 69.71 ± 19.69 | 72.29 ± 18.65 | 0.178 |
| SUA (μmol/L) | 303.75 ± 88.45 | 265.70 ± 91.32 | < 0.001* | 352.44 ± 95.76 | 306.54 ± 85.44 | < 0.001* |
| AST (U/L) | 22.57 ± 11.51 | 19.35 ± 6.73 | 0.001 * | 24.98 ± 17.50 | 20.49 ± 10.21 | 0.002* |
| ALT (U/L) | 33.13 ± 23.96 | 23.45 ± 15.77 | < 0.001* | 32.26 ± 19.34 | 20.77 ± 12.40 | < 0.001* |

******p* < 0.05 indicates statistical significance

BMI: Body Mass Index; WC: waist circumference; SBP: systolic blood pressure; DBP: diastolic blood pressure; HBG: high blood glucose; FBG: fasting blood glucose; TG: triglycerides; TC: total cholesterol; HDL: high density lipoprotein-cholesterol; LDL: low density lipoprotein-cholesterol; BUN: blood urea nitrogen; SCr: serum creatinine; SUA: Serum uric acid; AST: aspartate aminotransferase; ALT: alanine aminotransferase.
