# Supplementary material for: Association between the PNPLA3 I148M Polymorphism and Non-Alcoholic Fatty Liver Disease in the Uygur and Han Ethnic Groups of Northwestern China
Source: PLoS One. 2014 Oct 7;9(10):e108381. doi: 10.1371/journal.pone.0108381 (PMC4188522; doi:10.1371/journal.pone.0108381)
Supplement: Table S2 — Clinical and biochemical characteristics of participants, stratified by PNPLA3 I148M polymorphism status. (DOCX) [file pone.0108381.s002.docx]

Table S2 Clinical and biochemical characteristics of participants, stratified by PNPLA3 I148M polymorphism status

| Clinical index | Uyghur | | | *p* | Han | | | *p* |
| --- | --- | --- | --- | --- | --- | --- | --- | --- |
|  | CC | CG | GG |  | CC | CG | GG |  |
| Age(year) | 42.92±10.38 | 43.65±10.49 | 42.77±9.83 | 0.752 | 42.25±8.85 | 41.66±8.98 | 42.35±9.07 | 0.793 |
| BMI(kg/m2) | 24.68±3.53 | 25.15±4.45 | 24.29±4.14 | 0.320 | 26.69±4.07 | 26.73±4.36 | 27.16±4.14 | 0.775 |
| WC(cm) | 87.72±9.83 | 88.77±11.54 | 83.62±12.62 | 0.059 | 93.33±10.57 | 92.58±10.84 | 93.26±11.70 | 0.936 |
| SBP(mmHg) | 122.24±17.47 | 116.34±15.95 | 119.36±14.70 | 0.044***** | 118.49±14.73 | 121.75±16.47 | 122.41±18.49 | 0.142 |
| DBP(mmHg) | 74.30±12.50 | 76.53±11.78 | 78.76±11.93 | 0.035***** | 74.72±10.74 | 76.59±10.59 | 74.33±13.94 | 0.264 |
| HBG(g/L) | 143.94±14.26 | 145.01±14.50 | 144.26±15.78 | 0.827 | 142.26±17.31 | 143.99±16.93 | 139.16±20.99 | 0.216 |
| FBG(μmol/L) | 5.20±0.82 | 5.27±0.85 | 5.33±1.25 | 0.603 | 5.16±1.12 | 5.12±1.20 | 5.33±2.58 | 0.630 |
| TG(mmol/L) | 2.05±2.04 | 1.98±2.28 | 1.98±2.04 | 0.954 | 1.81±1.69 | 1.54±1.08 | 1.30±0.52 | 0.028***** |
| TC(mmol/L) | 4.74±0.82 | 4.85±0.98 | 4.65±0.87 | 0.300 | 4.80±0.93 | 4.84±0.96 | 4.88±0.98 | 0.877 |
| HDL(mmol/L) | 1.20±0.34 | 1.21±0.38 | 1.20±0.38 | 0.979 | 1.25±0.30 | 1.28±0.39 | 1.36±0.31 | 0.177 |
| LDL(mmol/L) | 2.80±0.72 | 2.86±0.80 | 2.79±0.71 | 0.724 | 2.98±0.75 | 2.97±0.68 | 2.97±0.78 | 0.996 |
| BUN(mmol/L) | 4.95±1.39 | 5.19±1.43 | 5.05±1.28 | 0.274 | 4.84±1.23 | 4.77±1.20 | 4.62±0.96 | 0.493 |
| SCr(μmol/L) | 72.95±22.55 | 70.67±17.75 | 67.40±13.06 | 0.169 | 68.69±17.36 | 70.27±21.60 | 65.30±12.90 | 0.225 |
| SUA(μmol/L) | 335.01±100.00 | 332.20±89.08 | 297.58±84.30 | 0.027* | 279.04±86.73 | 300.58±98.87 | 256.91±73.55 | 0.005***** |
| AST(U/L) | 22.68±18.13 | 22.45±11.28 | 23.23±12.98 | 0.938 | 20.81±10.91 | 21.49±8.70 | 20.21±8.42 | 0.650 |
| ALT(U/L) | 24.62±16.49 | 26.52±16.36 | 29.67±20.50 | 0.162 | 27.02±20.39 | 30.88±23.42 | 25.31±12.78 | 0.118 |

^*^*p* < 0.05 indicates statistical significance

BMI: Body Mass Index; WC: waist circumference; SBP: systolic blood pressure; DBP: diastolic blood pressure; HBG: high blood glucose; FBG: fasting blood glucose; TG: triglycerides; TC: total cholesterol; HDL: high density lipoprotein-cholesterol; LDL: low density lipoprotein-cholesterol; BUN: blood urea nitrogen; SCr: serum creatinine; SUA: Serum uric acid; AST: aspartate aminotransferase; ALT: alanine aminotransferase.
